# Cesarean Myomectomy: Reflections on Clinical and Surgical Controversies between a New Trans-Decidual Technique vs. Traditional Method

**DOI:** 10.3390/medicina60040609

**Published:** 2024-04-08

**Authors:** Radmila Sparić, Luka Andrić, Oguz Guler, Antonio Malvasi, Ivana Babović, Safak Hatirnaz, Miriam Dellino, Andrea Tinelli

**Affiliations:** 1 Faculty of Medicine, University of Belgrade, Dr Subotića 8, 11000 Belgrade, Serbia; ivana.r.babovic@gmail.com; 2 Clinic for Gynecology and Obstetrics, University Clinical Centre of Serbia, Dr Koste Todorovića 26, 11000 Belgrade, Serbia; lukaandric9@gmail.com; 3 Department of Obstetrics and Gynecology, Private Asya Hospital, Yenimahalle mh. 537, St. No.5 Gaziosmanpasa, 34250 Istanbul, Turkey; oguz_gulers@yahoo.com; 4 Department of Interdisciplinary Medicine (DIM), University of Bari, Aldo Moro, 70100 Bari, Italy; antoniomalvasi@gmail.com (A.M.); miriamdellino@hotmail.it (M.D.); 5 Mediliv Medical Center, Kale, Mevlevihane Cd. No.11, 55100 Samsun, Turkey; safakhatirnaz@gmail.com; 6 Department of Obstetrics and Gynecology, CERICSAL (CEntro di RIcerca Clinico SALentino), “Veris delli Ponti Hospital”, Via Giuseppina Delli Ponti, 73020 Scorrano, Italy; andreatinelli@gmail.com

**Keywords:** cesarean myomectomy, uterine fibroids, uterine myoma, cesarean section, pregnancy, complications, fertility

## Abstract

Up to 70–80% of women of reproductive age may be affected with the most common uterine tumors, known as fibroids or myomas. These benign tumors are the second most prevalent cause of surgery among premenopausal women. Predictions show that the occurrence of myomas in pregnancy will increase, and that the risk of having myomas during pregnancy increases with advanced maternal age. Although most women with fibroids do not experience any symptoms during pregnancy, up to 30% of women experience problems during pregnancy, childbirth, and the puerperium. The viability of myoma excision during cesarean surgery (CS) is a contentious issue raised by the rising incidence of myomas in pregnancy and CS rates. A new surgical procedure for removing fibroids using a trans-endometrial approach, which involves making an incision through the decidua itself, has put into doubt the long-standing practice of cesarean myomectomy (CM) with a trans-serosal approach. Some authors have recently advocated for this last approach, highlighting its advantages and potential uses in real-world situations. The purpose of this paper is to critique the present approach to cesarean myomectomy by analyzing the clinical and surgical distinctions between the two approaches and providing illustrations of the CM methods.

## 1. Introduction

Myomas, leiomyomas or fibroids represent the most common benign tumors of female genital organs. Fibroids can affect up to 70–80% of women who are fertile, of whom 40% exhibit symptoms [1,2], representing also the second most common reason for surgery in premenopausal period [1]. Women are affected by fibroids mainly during the reproductive period, and in certain cases, mass symptoms or suspected leiomyosarcoma may need treatment even after menopause [3]. Operation by myomectomy or hysterectomy is the only treatment that can be used to treat fibroids [2], and both these procedures represent major surgery. Nevertheless, because fibroids can recur during the reproductive age, some patients may need repeated surgery [4].

Significant direct and indirect costs to the healthcare system are linked to fibroids [2], including treatment expenses, decreased productivity at work, and negative effects on women’s health-related quality of life (HR-QoL) during the reproductive years [1]. Treatment of symptomatic fibroids leads to improvement in HR-QoL, both in relation to physical and mental functioning [1].

Among other reasons, waiting until a woman reaches her late reproductive age raises her chance of getting fibroids during her pregnancy. Future projections indicate that this incidence will increase, particularly in cases of very advanced maternal age [5]. It is believed that 25% of women having in vitro fertilization with donated oocytes present fibroids in pregnancy [6], and even while most fibroids do not produce any symptoms throughout pregnancy, up to 30% of women encounter issues with pregnancy, birth, and the puerperium [5].

It is already known that the number of cesarean sections (CSs) performed worldwide is rising, and this trend is predicted to continue, especially for myoma-affected women, who are often in their late reproductive years [7]. It is estimated that women with myomas have a 27% increase in the cesarean birth risk [8]; therefore, it is necessary to seriously think about the possible removal of fibroids at the same time as CS.

Recently, a new surgical technique known as endometrial myomectomy (EM) entered the obstetrician’s toolkit. It can also be performed through the uterine cavity and has gained quite a popularity among obstetricians [9,10]. Unlike all the previously described methods, the fibroid is dissected not by incision through uterine serosa, but through incision made through the decidua itself. The rationale of the new technique called endometrial myomectomy (EM) is that it might be more favorable both in relation to short-term and long-term postoperative outcomes. Proponents of this method claim that overall hemorrhagic risks are reduced without causing significant differences in terms of other postoperative outcomes. Furthermore, there are hypotheses that suggest it could be useful in some situations where performing standard CM is highly discouraged or even unfeasible. In order to determine the ideal location for the novel procedure in contemporary obstetric surgery, we made the decision to examine the literature in order to assess the State of the Art regarding EM and compare it with the traditional method of serosal myomectomy (SM).

## 2. Materials and Methods

We conducted a comprehensive review of Scopus and PubMed databases from 1990 to January 2024 to identify available data concerning the EM. A combination of keywords, such as “cesarean myomectomy”, “caesarean myomectomy”, “serosal myomectomy”, “trans endometrial myomectomy”, “endometrial myomectomy”, “fibroid”, “myoma”, “leiomyoma”, “cesarean section”, “caesarean section”, “surgical complications”, “pregnancy”, “fertility”, and “surgical outcome” were searched. We included only peer-reviewed articles involving human subjects. Additional articles were identified through cross-referencing. All evaluated papers focused on patients aged between 18 and 50 years, encompassing reproductive age to pre or perimenopause status. Authors excluded papers that were not related to the CM topic. The purpose of this paper is to critique the present approach to cesarean myomectomy by analyzing the clinical and surgical distinctions between the two approaches and providing illustrations of the CM methods.

## 3. Trans-Endometrial Cesarean Myomectomy in Common Clinical Practice

**Figure 1:**
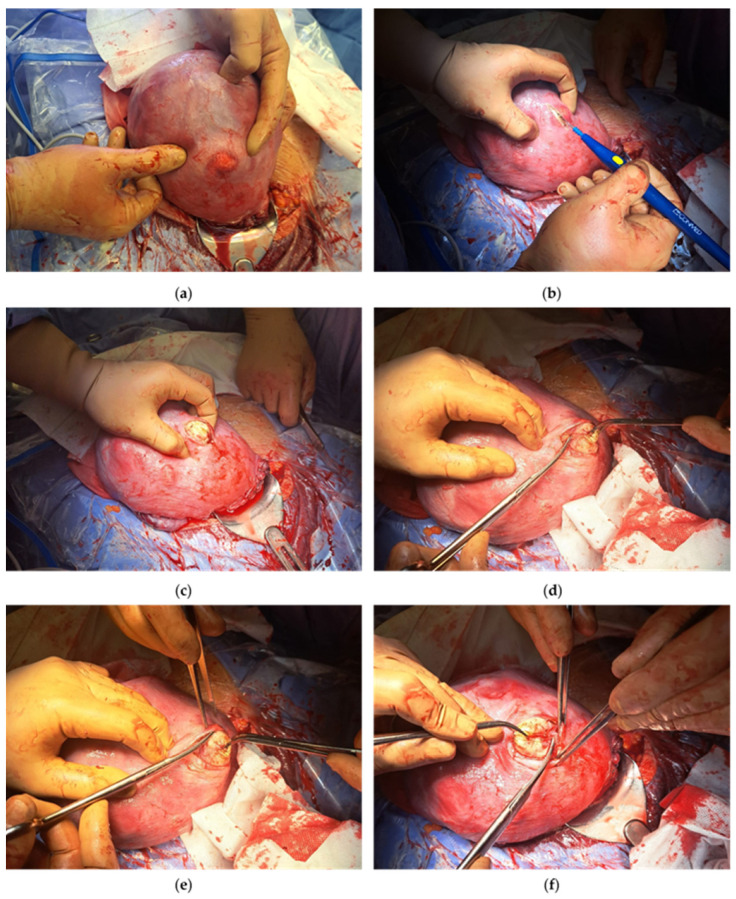

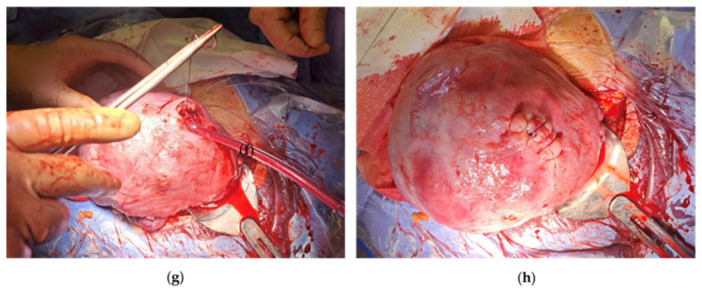
Enucleation of fibroid in anterior uterine wall via conventional serosal approach. (**a**) Identification of 3 cm leiomyoma on anterior uterine wall (FIGO type 5); (**b**,**c**) serosal incision via electrocautery and pushing leiomyoma onto surface; (**d**–**f**) intracapsular sharp dissection; (**g**) endometrial incision suture; (**h**) final view of sutured endometrial incision.

**Figure 2:**
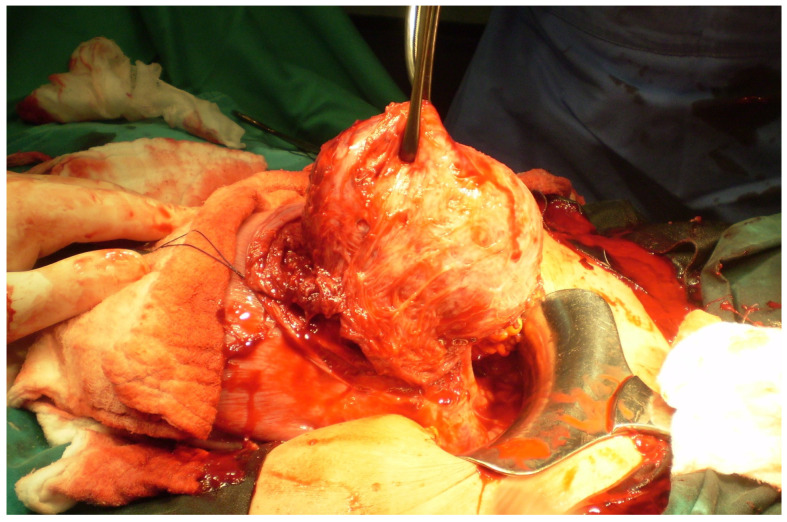
Evacuation of myoma previa through lower uterine segment incision.

Since the advent of myomectomy in clinical practice, the conventional wisdom in myoma surgery has been to perform a SM wherever possible without entering the uterus. That was the situation prior to the development of hysteroscopic surgery, which gained popularity as a treatment for fibroids with a minimum volume of 50% that protruded into the uterine cavity and were pedunculated or submucosal [11], until the hysteroscopic approach was suggested in some cases for fibroids that made up less than half of the entire volume of the uterine cavity [12]. For many years, CM was carried out in the same way: most fibroids were removed by dissection through the uterine serosa as a SM (Figure 1), while pedunculated submucosal myomas were removed by clamping, cutting, and suturing the pedicle in the uterine cavity. Myomectomy is usually performed after the hysterorrhaphy, with the exception of pedunculated submucosal myomas or myomas interfering with uterine incision, where CM is occasionally necessary to make the requisite space for hysterotomy, fetal extraction, and uterine suturing (Figure 2).

**Figure 3:**
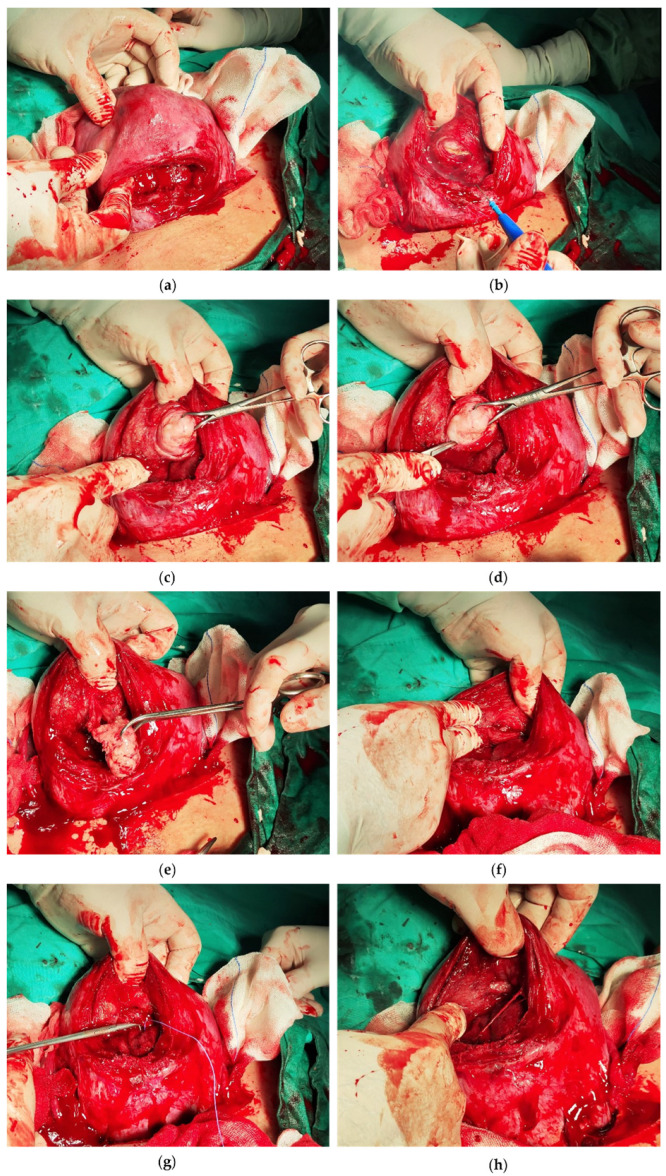
Enucleation of fibroid in anterior uterine wall via trans-endometrial approach. (**a**) Identification of 4 cm leiomyoma on anterior uterine wall (FIGO type 5); (**b**) trans-endometrial incision via electrocautery; (**c**) pulling leiomyoma with Backhaus towel forceps; (**d**) intracapsular blunt dissection; (**e**) removing leiomyoma; (**f**) view of intracapsular space; (**g**) single layer unidirectional running suture; (**h**) final view of sutured endometrial incision.

The lower uterine segment (LUS) incision and the myoma bed are typically sutured using the same stitches in case of myoma previa. In contrast to SM, which is referred to as standard CM, EM is a new surgical strategy for CM that has just recently been documented in the literature on obstetric surgery. This was initiated in 2018 by two distinct surgical groups from Taiwan and Turkey who published their outcomes using this treatment [9,10]. The hysteroscopic myomectomy technique spurred the idea for the EM approach since it reduces the risks associated with standard SM, specifically those of hemorrhage and the formation of intrabdominal adhesions [9,10,13]. In a nutshell, the procedure involves trans-endometrial incision, pseudocapsule sparing technique enucleation of the fibroids, clamping and ligation of all vessels at the base of the myoma fovea, and suturing of the myoma bed using Vicryl 1 interrupted stitches. When additional hemostasis operations are required, the authors recommend using what are known as “figure of eight” stitches [14]. The endometrial defect was sutured only in cases when it was larger than 30 mm (Figure 3). Fibroids close to the uterine LUS hysterotomy are generally suggested to be removed by incision between endometrium and myometrium [9].

Regardless of size, the goal of EM is to eliminate fibroids that are closer to the endometrial cavity by taking advantage of uterine physiology. This s enables intramural fibroids during CS to be mobilized near to the endometrium and readily removed by trans-endometrial incision. Theorized by the pioneers of the EM technique, this novel approach minimizes blood loss without extending the surgical time by preserving the integrity of the uterine tissues throughout CM [14]. Uterine involution reduces the size of the surgical site, obliterates intramyometrial death spaces, and provides a hemostatic effect by squeezing the blood vessels. Furthermore, the myoma enucleation through trans-endometrial incision is associated with an overall smaller size of the incision when compared to SM, as affirmed by the pioneers of the EM technique [14,15]. Since only spiral arteries are harmed, removing a fibroid from the endometrial surface should carry a far lower chance of major uterine artery lesions. These arteries will narrow rapidly during uterine contractions. Furthermore, the physiological uterine involution that occurs throughout the puerperium should reduce the risk of hemorrhaging during and after surgery [14,15]. Another possible benefit of EM should include, according to the literature data, the possibility to use EM in cases of cornual and posterior wall fibroids, as well as intramural fibroids [14]. Finally, EM should reduce the unfavorable effects of SM on peritoneal surfaces, possibly reducing the chances of adhesion formation and the consequent detrimental effect on successive reproductive performance [14]. EM is associated with reduced incisions and fewer sutures on the uterine serosa [16]. Positive effects of EM, in relation to adhesion formation, were documented in the literature [10,16]. As the authors of the new method state, EM seems like a reasonable option for myomas close to the LUS incision site, which cannot be excised through LUS incision [15]. Conversely, EM could be the recommended technique to remove fibroids during CS if the pregnant woman has pelvic adhesions, which make it difficult to remove the uterus from the abdominal wall, for obvious reasons [17]. EM has no stated contraindications, but it should not be offered in women with a history of multiple trans-endometrial surgeries, a history of Asherman syndrome, placental adhesion abnormalities, and possibly in cases with a history of thin endometrium. Furthermore, EM can be used for fibroids that are not in a serosal or subserosal site and, during a CS, several fibroids could also be eliminated. Theoretically, both EM and SM can be used in conjunction for numerous fibroids, such as serosal or pedunculated myomas.

## 4. Investigations on Endometrial Cesarean Myomectomy

Because now there are only a few studies comparing the two techniques, and with a small number of patients to be able to draw definitive conclusions, we have summarized what is present in the literature in Table 1.

Hatirnaz et al. [9] compared 22 cases of EM with 24 matched cases of SM in women having symptomatic submucosal or intramural fibroids of the uterine anterior wall. Forty days following the EM, all patients in this group had saline infusion sonography (SIS) to evaluate endometrial damage and detect intrauterine adhesions. The groups were comparable in terms of age, size, and myoma site (mainly intramural). The duration of the myomectomy procedure and the amount of surgical bleeding varied, nevertheless. The EMs resulted in 165 mL less blood loss and lasted, on average, 8 min less than the SMs. The authors deduced from their findings that, in comparison to SM, EM is linked to reduced blood loss and a shorter duration of surgery, but it also carries no higher risks of extended hospital stays or the development of intrauterine adhesions following the treatment. Furthermore, there is presumably less likelihood of intraabdominal adhesions forming because the uterine serosa is still intact.

Another Turkish research group investigated EM in women having single intramural myomas on the anterior uterine wall in a study group of 41 patients versus 52 patients who underwent traditional SM [16]. The study was aimed at comparing the outcomes of two myomectomy procedures and evaluating the long-term consequences of adhesions encountered during the subsequent CS and subsequent pregnancies. This paper provided a detailed description of the EM technique, which is similar to the technique employed by other authors [9,10]. Concerning baseline clinical and demographic parameters, all 93 subjects were similar. In terms of myoma diameter, the SM group had a larger average, but there was no discernible difference between them and the EM group (63.6 mm against 50.5 mm, respectively). Total duration of the surgery was significantly different between the groups, as CS with EM lasted on average 50.5 ± 10 min while CS with traditional SM lasted 63.6 ± 15.2 min. Length of hospital stay, preoperative and postoperative hemoglobin values and hemoglobin difference, frequency of blood transfusions, and incidence of postoperative fever were similar between the groups. Following the CM procedure, 17 patients after EM and 14 after SM underwent repeated CS. Both patient groups were comparable when it came to gestational week, time interval between CSs, neonatal birth weight, Apgar ratings, and rates of admission to the neonatal intensive care unit (NICU). Concerning adhesion scores recorded during repeated CS using the Ichikawa et al. [19] score, significantly lower adhesion scores were registered in women who had EM compared to those who had SM. Furthermore, Yıldırım Karaca et al. [16] underlined that EM represents a safe and feasible surgical option for intramural fibroids in relation to maternal and neonatal postoperative morbidity, with shorter operative time when compared to SM. Cited authors were the first to register lower postoperative adhesion scores during repeated CS in women who underwent EM.

Huang et al. [10] described a group of 63 pregnant women who had EM during their initial CS and were scheduled for a repeat CS later. The patients’ obstetric and surgical outcomes were assessed. These authors stated that exteriorization of the uterus and clamping the edges of the LUS incision to produce transient hemostasis were included in EM. The uterus was grasped in the left arm once the fibroid was identified and crushed from the serosa into the uterine cavity, while a thumb was used to hold the upper border of the LUS incision. A linear incision through the endometrium was performed with a scalpel or monopolar electro scalpel at 30 W once the myoma protruded into the endometrial cavity. Army-navy retractors were used to expose the myoma which was then hooked and enucleated from its pseudocapsule by blunt or sharp dissection. Myometrial defects were sutured in a single layer using interrupted 1–0 Vicryl stitches. Endometrial defects were not sutured in all cases without apparent bleeding. Thirty-eight of the sixty-three women who were included in the study (63%) had two or more myomas removed, with an average diameter of 76 ± 22 mm. There was a 3.7 ± 1.1-year gap between CSs. Neonatal weight was also considerably larger (3188.6 versus 2796.4 gr, respectively) in connection to obstetric outcomes; gestational age was also greater during following pregnancy (38.5 against 36.5 weeks, respectively). Additionally, the first pregnancies had a substantially greater prevalence of spontaneous preterm births (30.2% versus 7.9%). There were no cases of uterine rupture and placenta accreta, while the occurrence of placental abruption and placenta previa was comparable between the groups. In relation to surgical outcomes, i.e., blood loss, blood transfusion, postoperative fever and duration of hospitalization, they were similar. Only the duration of the operation was significantly shorter for the subsequent CS (41.5 ± 9.2 versus 46.7 ± 7.1 min, respectively). Mean adhesion scores calculated using a modified American Fertility Society scoring system were similar [20,21].

A later multicentric study conducted by a Turkish research team involved 360 women with myomas, of which 118 were part of a study group that received EM. The two groups serving as controls were 120 women who had standard SM and 122 women who had CS alone [14]. Patients who had CM had anterior, posterior or cornual fibroids type 2–5. In relation to the age, gravidity, parity, BMI, gestational age at delivery, frequencies of previous preterm births and myomectomies, as well as indications for CS, all three groups were similar. The number of fibroids enucleated and their sizes were comparable in the EM and SM groups, and most of them in the entire study were intramural. Nevertheless, most myomas in the SM group were subserosal, while most of the myomas in the EM group were hybrid (33.1% and 17.5%). SM lasted significantly longer than EM (13.85 versus 8.17 min, respectively), and it was associated with a significantly prolonged duration of CS (46.53 versus 37.88 min, respectively). Postoperative hemoglobin values were significantly lower in the SM group. In relation to frequency of uterine atony, there were no significant differences throughout the groups.

CM outcomes in pregnant women affected by intramural fibroids greater than 80 mm in diameter were investigated by Shi et al. [15]. The authors evaluated the safety and feasibility of CM in a total of 190 women with fibroids type 3–5, according to the FIGO classification system [22,23]. Out of those patients, 130 women underwent CM and the other 60 patients having only CS were the controls. EM was performed on 64 patients, and SM on 66 patients. In thirty-three cases, SM was used before LUS suture, and in thirty-three cases, SM was used after LUS suture. After uterine exteriorization, a tourniquet was used to execute all CMs on the lower portion of the uterus. Moreover, the authors used 1 g of tranexamic acid and 250 mg of carboprost to increase uterine contraction in situations of severe bleeding. If the outcomes were not satisfactory, the uterine artery’s ascending branches were sutured and the uterus was ballooned. The study groups had comparable baseline features, such as the average size of the largest fibroid, which measured 90 mm. Both the number and the location of fibroids were similar. The perioperative outcomes of the CM were mainly favorable, with significant differences in terms of operation time, pre-and postoperative hemoglobin drop, and intraoperative blood loss between CM groups and the control group. Although postpartum hemorrhage, blood transfusions, and uterine artery ligations were more frequent in the CM group, there were no statistical differences between the CS only group and the CM groups. Time required for myoma enucleation was shorter, and hemoglobin drop and blood loss were reduced in patients who underwent EM with myomas type 3 and type 5. In cases of myoma type 4, there were no significant differences in terms of those outcomes between EM and SM. The authors concluded that although EM may cause greater myometrial tissue damage than SM, it is not linked to better results in women with type 4 myomas in terms of the amount of time needed for fibroid excision and intraoperative blood loss.

Wang et al. [17] settled a retrospective cohort study including patients with intramural fibroids of the posterior uterine wall larger than 30 mm. Fifty patients with EM were included in the study group, while 48 patients with SM were in the control group. Based on their diameter, fibroids were divided into three groups: ≥30 ˂50 mm, ≥50 ˂100 mm, and ≥100 mm. Clinical and demographic baseline features of the patients were similar. The characteristics of the fibroids did not differ significantly between both groups: the majority of the fibroids in both groups were larger or equal to 50 mm and smaller than 100 mm (80.0% in the EM group and 83.3% in the SM group, respectively), with most of them located in the uterine corpus (96.0% in the EM group and 93.8% in the SM group, respectively). The median size of the myomas was 70 mm. The maximum size was 150 mm in the EM group and 130 mm in the SM group, respectively. Total operative time was significantly shorter in the EM group, postoperative hemoglobin drop was significantly reduced in the EM group, and the estimated blood loss was smaller in the EM group. Reported incidence of intraoperative hemorrhage was 4.0% in the EM group and 8.3% in the SM group.

## 5. The Future of Trans-Endometrial Myomectomy in Modern Obstetric Surgery

Despite five available meta-analyses on CM, none of those addresses the topic of EM [24,25,26,27,28]. The CM technique through an internal trans-endometrial (or decidual) approach, EM, which improved obstetric outcomes of subsequent pregnancies without any so far registered long-term adverse surgical outcomes, was the subject of a scientific debate immediately after the publications by Hatirnaz et al. [9] and Huang et al. [10].

This debate raised several important questions, the most important of which was whether we should adhere to the old dogma about avoiding CM in all pregnant patients [29]. According to Pandey [30], this innovative surgical technique might improve the obstetric outcome of a subsequent pregnancy without having an adverse surgical outcome in the short- or long-term. He did stress, nevertheless, that evaluating additional risk factors for unfavorable obstetric outcomes will produce higher-quality data on the results of the subsequent pregnancies. In response, the authors stressed that there were no visible anomalies or morphological changes to the uterine cavity seen during the hysteroscopic evaluation of the patient who had received EM [18].

Olah et al. [29] declared that the outdated advice to “*avoid myomectomy in pregnancy at all costs, including during CS*” should be abandoned because certain cases can be treated by CM without experiencing appreciable additional morbidity. He expressed his opinion that additional evaluation of novel surgical techniques in modern obstetric surgery is necessary because of the physiological changes that pregnancy brings about in the uterus. Many authors emphasized that because CM requires a smaller uterine incision than a later interval myomectomy, it is a more feasible choice [31].

Hatirnaz et al. [14] underlined that the benefit of EM is an even smaller incision in the uterus compared to SM, which becomes even smaller with time, due to physiological uterine contractions which continue until the full involution of the uterus. Moreover, Wang et al. [17] underlined that EM is more suitable for larger fibroids as the remaining muscle layer surrounding those becomes thinner, which makes easier the protrusion of fibroids into the uterine cavity, facilitating fibroid enucleation through endometrial incision. On the other hand, they proposed that trans-endometrial resection and the removal of smaller fibroids through squeezing into the uterine cavity are linked to nearly the same depth of myometrial incision as for SM. According to the authors performing EM procedures, the risk of endometrial injury is comparable to the risk when performing hysteroscopic myomectomy or polypectomy [10,12,13,14]. Initial data about subsequent pregnancies after EM claimed to be promising [10,16]. The puerperium’s decidua peels may hinder adhesion formation, which could be the reason why there is not any data about intrauterine adhesions following EM to date [15]. Neither Hatirnaz et al. [9,14] nor Shi et al. [15] found any intrauterine adhesions following EM. Patients were examined for their presence via SIS 40 days after the CS in one study and using B-mode ultrasonography 42 days after the procedure in another [9,15]. It is hypothesized that SM performed through a large and vascular serosal surface of the uterus could be more prone to bleeding than incision performed via trans-endometrium [14]. Available literature data endorse the theory that EM is associated with reduced hemorrhage when compared to a traditional SM approach. Hemorrhage after CM is sometimes intractable and requires cesarean hysterectomy to stop it [32]. Decreased hemorrhage during EM can be scientifically documented by lower blood loss, transfusion rates, and hemoglobin concentrations [9,10,14,15,16,17]. Bearing in mind that CM is considered to be a challenging procedure, primarily due to the risk of uncontrollable hemorrhage, the idea of trans-endometrial approach as a way to reduce this risk sounds very promising. Nevertheless, a small number of the available studies do not provide sustainable evidence yet for its wide acceptance. On the other hand, in the light of the growing number of women of reproductive age affected by fibroids and undergoing CSs, further investigation into this new technique is mandatory. Although not underlined by its promoters, it has an important additional benefit over the other techniques suggested to reduce hemorrhage risks [31]. In other words, it does not call for any form of long-term uterine devascularization through blood vessel ligation or embolization, which could be harmful to subsequent pregnancies. Devascularization of the uterine or hypogastric arteries is a documented technique to lessen bleeding, but it is questionable why women of reproductive age should undergo such a procedure given the potential consequences for future fertility and pregnancy outcomes. Performing CM through the uterine cavity could reduce the risks of abdominal adhesion formation and consequent complications caused by adhesions [16,19]. Nevertheless, so far, reliable data regarding the effects of EM on adhesion occurrence between the uterus, ovaries and Fallopian tubes, with detrimental effects on fertility after CMs are mainly missing. In addition, data on the effects of the procedure on the formation of abnormally invasive placenta (AIP) and other placental pathologies, as well as uterine rupture in subsequent pregnancies, are lacking. The safety and feasibility of EM requires more investigation to define the incidence and risks of possible late complications of CM related to this technique. Yildirim Karaca et al. [16] underlined that EM is not suitable for fibroids located far from the LUS incision site and subserous fibroids. Unlike Hatirnaz et al. [14], they suggested that EM is not an option for cornual fibroids and fibroids of the posterior wall of the uterus [16]. Later publications suggested EM as a preferred method for CM in the case of posterior uterine wall intramural fibroids [17]. Now, EM is claimed to provide the opportunity to remove fibroids inaccessible by a serosal approach, i.e., cornual, deep intramural, and those located in the posterior uterine wall [14,16]. Further research is necessary to validate this concept, as cornual fibroids are typically left in place due to the possibility of both severe bleeding and tubal blockage.

## 6. Conclusions

The issue still merits scientific discussion even though the research that is currently available provides positive information about the safety and viability of CM in addition to information on possible major issues with the so-called “no touch” CS in myoma-affected women. Thus, the use of the EM technique in obstetric surgery did not settle the century-old controversy on the justification of CM. On the other hand, it offered a new angle on a long-standing debate: which is better—the trans-endometrial or the serosal method? The use of EM in cases of intramural fibroids sparked debate on the subject, and rightly so, as there is an absolute dearth of information concerning fibroids in the fundal region. Whether SM or EM is employed, the same surgical outcomes—bleeding and the need for blood transfusions, a lengthier recovery time, and an extended hospital stay—are discussed. Regarding long-term morbidities, there is a lack of information on adhesion development, myoma recurrence, uterine scar integrity, and abnormal placentation. This procedure would differ greatly from the usual one employed up to this point; therefore, large, well-planned studies will be needed in the future to evaluate the ideal surgical strategy in cases with multiple CMs conducted combining EM and SM.

## Figures and Tables

**Table 1:** Available studies on endometrial myomectomy.

Author, Year of Publication, Reference Number	Study Population		Myoma Characteristics				Main Outcomes	Other
	Study Group	Control Group	Size	Type	Localization	FIGO Type		
Hatirnaz et al., 2018 [9]	22 EM	24 SM	57.83 ± 12.53 mm in the study and 59.59 ± 12.74 mm in the control group.	Mainly intramural, less subserosal + intramural, multiple one case in each group.	Anterior wall of the uterus	N/A	Duration of CM and amount of blood loss were significantly lower in EM cases.	N/A
Huang et al., 2018 [10]	63 EM with subsequent pregnancy	/	The mean size of myomas was 76 ± 22 mm. The mean number of removed myomas was 1.8 ± 0.8.	N/A	N/A	N/A	The mean GA at birth and newborn weight at the subsequent CS were superior to those at the first CS. Spontaneous preterm birth, SGA infants, and PPROM occurred more often in the first pregnancy.	Amount of blood loss, blood transfusion, postoperative fever, length of hospital stay, and adhesion score were similar across the two stages of CS. Postoperative hysteroscopy showed no major anatomical changes from the EM [18]
Hatirnaz et al., 2021 [14]	118 EM 120 SM	122 CS only	40 mm (20–110) in EM group and 40 mm (20–100) in SM group.	Mainly intramural; less hybrid (FIGO type 2–5) and subserosal.	N/A	The frequency of type 2–5 myomas was higher in the EM than in the other two groups; the frequency of type 5 myomas was higher in the SM than in the other two groups.	Decline between pre- and postoperative hemoglobin concentrations was significantly higher in the SM group than the other two groups. Duration of surgery was significantly longer in SM than in other two groups.	Patients who had EM underwent SIS at the 6th postoperative week, and no intrauterine adhesions were determined within the uterine cavity.
Yıldırım Karaca et al., 2021 [16]	41 EM	52 SM	57 ± 35 mm in study group and 44 ± 17 mm in control group.	All myomas were intramural.	In myomas located far from the incision line, subserosal and myomas in posterior wall, SM was performed.		Duration of surgery was shorter in the EM group. Patients in the EM group had significantly lower adhesion scores in their subsequent pregnancy.	No difference in length of hospital stay, hemoglobin difference, blood transfusion requirement and postoperative fever.
Shi et al., 2023 [15]	64 EM 66 SM (33 SM before suturing UI and 33 SM after suturing UI)	60 CS only	90 mm (80–150) in EM group, 90 mm (80–170) in SM group and 90 mm (80–180) in control group. Number of removed myomas is 1 (1–5) in EM, 2 (1–5) in SM, and 1 (1–4) in control group.	All myomas are intramural.		FIGO type 3 to 5	Surgery duration, postoperative exhaust time and blood loss were significantly lower in the control group than in the study groups, as well as in the EM than in the SM group for type 3 and 4 myomas.	No significant differences were found in the incidence of PPH, transfusion, uterine artery ligation, intrauterine adhesions, 5 min Apgar score and asphyxia between the two study groups.
Wang et al., 2023 [17]	50 EM	48 SM	70 mm (30–150) in study group and 70 mm (30–130) in control group.	All myomas are intramural.	Posterior wall	N/A	Duration of CM and amount of blood loss were significantly lower in EM group.	The postoperative ventilation time was significantly longer in the SM group.

Abbreviations: EM = endometrial myomectomy; SM = serosal myomectomy; CM = cesarean myomectomy; GA = gestational age; CS = cesarean section; SGA = small-for-gestational-age; PPROM = preterm premature rupture of membranes; SIS = saline infusion sonohysterography; UI = uterine incision; PPH = postpartum hemorrhage.

## Data Availability

Not applicable.
